# Examining Benchmarks of Sexual Recidivism Rates for Short, Moderate, and Long-Term Follow-Up Periods: A Meta-Analysis of Canadian and American Studies

**DOI:** 10.1177/15248380251338791

**Published:** 2025-06-03

**Authors:** Patrick Lussier, Evan McCuish, Elisabeth St-Pierre, Arthur-Lou Baguet

**Affiliations:** 1 Université Laval, Quebec, QC, Canada; 2 Centre International de Criminologie Comparée and the Institut National de Psychiatrie Légale Philippe-Pinel, Montreal, QC, Canada; 3 Simon Fraser University, Burnaby, BC, Canada

**Keywords:** base rate, follow-up, long-term, meta-analysis, risk assessment, sexual recidivism

## Abstract

Measuring sexual recidivism involves both a behavioral and a temporal component. The behavioral component is sexually reoffending, generally measured using official sources. The temporal component is the follow-up period during which sexual recidivism is examined. Research has shown that if the length of the follow-up period is extended, rates of sexual recidivism increase. What is less clear is the functional form of this relationship. The present study examines this relationship through a meta-analysis of 468 sexual recidivism studies conducted in Canada and the United States and published since 1940. The weighted pooled mean recidivism rates ranged from 0.06 (95% CI [0.05, 0.09]; mean follow-up of less than 3 years) to 0.17 ([0.12, 0.23]; mean follow-up of 12 years or more). These benchmarks should be used with caution given the wide variability of recidivism rates observed in studies with similar mean follow-up periods. Such caution is especially needed in when communicating the risk of recidivism over longer-term follow-up periods given the limited number of such studies.

## Introduction

Over the past two decades, assessing the risk presented by justice-involved individuals with a history of sexual offending has become an integral part of the criminal justice system (e.g., Kelley et al., 2020; Rettenberger & Craig, 2020; Rettenberger & Eher, 2024). The practice of risk assessment responds to long-standing concerns that such individuals remain at risk of sexual recidivism over a long period (e.g., Langevin et al., 2004; Prentky et al., 1997). Such risk assessments rely on available information about recidivism, such as the base rate of sexual recidivism, as well as risk assessment tools. These assessments can be pivotal in that they can influence both front-end designations (e.g., as a Sexually Violent Person in the United States or a Dangerous Offender in Canada) and back-end sentencing decisions (e.g., parole decisions, community supervision) with potentially long-term implications for justice-involved individuals (e.g., Sreenivasan et al., 2003). Over the past two decades, various policies, measures, and programs involving strict conditions and restrictions have been implemented in Canada and the United States in attempts to prevent justice-involved individuals from sexually reoffending (e.g., Cohen & Jeglic, 2007; Levenson & D’Amora, 2007; Lieb et al., 1998; Lussier, Chouinard et al., 2024). Practitioners who conduct risk assessments are required to make informed decisions that can have long-term effects for the individual involved. While risk assessment cannot determine whether justice-involved individuals will sexually reoffend, taking the base rate of sexual recidivism into account can help establish risk probabilities with a degree of confidence that outperforms unstructured clinical judgment (e.g., Hanson & Morton-Bourgon, 2009). These risk probabilities are established by examining data based on samples of justice-involved individuals with histories of sexual offending who have been followed for a defined period after community re-entry. However, short-term recidivism studies (i.e., 5 years or less) are overrepresented in the scientific literature and tend to underestimate the long-term risk of recidivism (e.g., Soothill & Gibbens, 1978; Soothill et al., 1976). Extrapolating long-term risk estimates from short-term recidivism studies could be misleading given that little is known about how risk estimates evolve over time, especially given that risk decreases as an individual ages through adulthood (e.g., Craig, 2008; Lussier & Healey, 2009). It is also unclear whether long-term recidivism studies are representative of the risk probabilities of recidivism for all justice-involved perpetrators, given the tendency for such studies to rely on small, biased, clinical-based samples. This may be especially true for studies with a lengthy follow-up period. At the very least, it is possible that studies with lengthy follow-up periods may have other unique methodological characteristics that should be considered as part of the practice of communicating risk of recidivism. These under-addressed methodological issues create significant problems for risk assessors in attempting to establish the risk of sexual recidivism, which can impact criminal justice decision-making in various contexts. The current study uses a meta-analytical framework that considers the length of the follow-up period while statistically adjusting for other methodological details to examine the base rate of sexual recidivism in Canada and the United States.

## Literature Review

Debates about the risk probabilities of individuals with histories of sexual offending have occurred for well over half a century (e.g., Guttmacher & Weinhofen, 1952; Tappan, 1951), contributing to the growth of this field of research. Such debate stems from attempts to answer a simple question: what is the risk of sexual recidivism? Risk probabilities are established by determining the proportion of individuals with histories of sexual offending who sexually reoffend over a certain time period. While researchers have been given the task to examine the likelihood of sexually reoffending (i.e., perpetrating another sexual offense), they have traditionally focused almost exclusively on information stemming from official sources (e.g., police data, court data) given the difficulties of obtaining valid and reliable information through other means and sources. There are at least four strategies for establishing the base rate of sexual recidivism. The first and perhaps most common strategy has been to empirically assess risk probabilities by examining data from a single sample of justice-involved individuals followed for a certain period after their community re-entry. A recent review of the literature found more than 800 empirical studies of sexual recidivism rates worldwide, with the percentage of sexual recidivists varying between 0 and 60 (Lussier, Chouinard et al., 2024). These varying results emphasize the importance of contextualizing risk probabilities by framing them as a series of questions dealing with who is at risk (e.g., age, sex, criminal record, prior incarceration), for what (e.g., type of sexual offending, seriousness, modus operandi, hands-on/hands-off), and in what context (e.g., responsivity to intervention, treatment participation/completion, community supervision; see also, Rice & Harris, 2006). Questions as to when sexual recidivism might occur have been seldomly raised even though they are imbedded in risk probabilities and are part of the responsibilities of risk assessors when engaging in case formulation to describe to the court the context in which a person might reoffend (Hart et al., 2011). Contextualization of risk probabilities that consider who, what, when, and in what context are difficult to establish using a single study sample, suggesting that a broader approach is needed. Three alternative strategies used to examine risk probabilities include comprehensive qualitative reviews, combining multiple samples, and conducting a systematic review and a quantitative meta-analysis.

The comprehensive qualitative review (e.g., narrative review, systematic review) of empirical study findings has not been used to establish risk probabilities per se but rather to describe, summarize, and contextualize the scientific literature (e.g., Furby et al., 1989; Greenberg, 1998; Lussier, Chouinard et al., 2024; Soothill, 2010). The authors of these reviews have stressed the difficulties summarizing the literature given the differences and methodological shortcomings of studies. Furthermore, qualitative reviews, while informative, have their own limitations, such as the difficulty in comparing rates across studies while simultaneously considering various methodological details. An alternative strategy has been to examine recidivism data after combining a small number of individual samples (e.g., Hanson et al., 2003) to attempt minimizing the limitations of single studies by combining various datasets, increasing sample size, and examining recidivism rates while adjusting for individual characteristics. While this approach is useful to address certain research questions, it is difficult to statistically control for study design characteristics and to examine the impact of methodological details on recidivism rates, especially if the study samples share similar limitations. Furthermore, it raises questions about how representative the combined samples are and whether the resulting findings can be generalized across samples, settings, and period. Conducting a recidivism study involves a series of methodologically oriented decisions (e.g., where to sample individuals, determining the sample size, identifying sampling criteria, selecting a measure of recidivism, determining what constitute a recidivism event), that can potentially create a series of biases (e.g., Webster et al., 2006). In order to minimize the methodological shortcomings and limitations of individual studies, others have relied on a systematic review and a meta-analysis of the literature (e.g., Caldwell, 2010; Cortoni et al., 2010; Hall, 1995; Lussier & McCuish, 2024; Lussier, McCuish et al., 2024). This approach, which involves combining recidivism rates and methodological details from individual studies, has not been used extensively to examine rates for various follow-up periods.

These four research strategies have contributed to the development of different narratives about risk probabilities and their relation to time. The first narrative is that sexual recidivism is to some degree a matter of time, as most justice-involved individuals can be expected to eventually sexually reoffend. On this interpretation, justice-involved individuals always remain at risk of sexual recidivism (e.g., Doren, 1998; Langevin et al., 2004; Prentky et al., 1997), which has fueled the development of stringent policies that affect justice-involved individuals and their families for extended periods after their release from prison (e.g., English, 1998). Proponents of this view argue that a lengthy follow-up period is needed for two main reasons: (a) the chance that an individual will be exposed to opportunities to commit a new sexual offense increases in relation to the length of time considered and (b) while an arrest is used as a measure of recidivism, there are often delays between the time a sexual offense occurs and an arrest is made (e.g., before a police complaint is made; see Mathesius & Lussier, 2014). This narrative assumes that all perpetrators of sexual offenses are characterized by a fixed and specific propensity (e.g., paraphilia) to commit sexual offenses, a propensity that is not always apparent in official information about recidivism because not all individuals who reoffend are detected by the criminal justice system (e.g., Abel et al., 1987; Groth et al., 1982; Langevin et al., 2004; Weinrott & Saylor, 1991). These arguments are, however, based on older samples/observations, questionable methodologies, and highly specific if not biased samples whose findings have not been consistently replicated (e.g., Smallbone & Wortley, 2004a,2004b; Webster et al., 2006). While official data, such as new arrests or convictions, underestimate actual sexual recidivism rates^1^ (e.g., Abbott, 2020; Lave et al., 2021; Scurich & John, 2019), the extent of this underestimation remains relatively unknown (Lussier, Chouinard et al., 2024).

The second narrative is that, despite unavoidable methodological issues and challenges, the base rate of sexual recidivism can be reliably estimated for specific follow-up periods and more conservative estimations of risk should be considered (e.g., Thornton et al., 2021). For example, Hanson et al. (2003) combined data from 10 samples from Canada, the United States, and the United Kingdom to examine risk probabilities in relation to sexual recidivism rates when the follow-up period is extended. They reported a 0.14 sexual recidivism rate for a 5-year period, 0.20 for a 10-year period, and 0.24 for a 15-year period, which suggests that the annual percentage of new recidivist events was approximately 2% to 3% over the first 5 years and dropped to about 1% thereafter. While the Hanson et al. (2003) study is well suited to addressing questions about risk probabilities for individuals (e.g., who represents a high risk of recidivism?), the limited number of samples considered makes it difficult to examine whether risk probabilities are influenced, at least in part, by methodological details (e.g., sample characteristics, measure of recidivism). Researchers working within the second narrative recognize that risk probabilities are group-based estimates and that there is significant within-group heterogeneity in terms of risk, treatment needs, and responsivity to treatment and intervention (e.g., Beggs & Grace, 2010; Craig et al., 2005; Mann et al., 2010; Olver et al., 2007; Proulx et al., 1997). This heterogeneity has an effect on the risk probabilities across samples and follow-up periods depending on the nature of the group/sample and the length of the follow-up period (e.g., Rice & Harris, 2006). Furthermore, it is unclear whether estimates published more than 20 years ago accurately reflect more current risk probabilities (Lussier & McCuish, 2024), especially because estimates published more than 20 years ago involved samples of persons adjudicated more than 30-35 years ago. Lussier et al. (2023) used a meta-analytic approach that included over 200 independent samples to provide updated information about sexual recidivism rates for different mean follow-up periods. Their findings, based on a different methodology than the one used by Hanson et al. (2003), reported lower estimates of risk, with a weighted mean sexual recidivism rate of 0.06 for studies with a mean follow-up period of 3 years or less, 0.09 for studies with a mean follow-up of less than 8 years, and 0.15 for studies with a mean follow-up period of at least 8 years. They also report significant heterogeneity of these rates in each of these follow-up periods, suggesting that other factors influence sexual recidivism rates beyond the length of the follow-up period.

While we can safely conclude that cumulative sexual recidivism rates increase as the follow-up period is extended, it has also been observed that desistance is the norm for perpetrators of sexual offenses (Lussier & Healey, 2009; Lussier et al., 2016). This third narrative challenges the view that risk probabilities are fixed over a life course, irrespective of age (Barbaree et al., 2003). Researchers have been aware for several decades that an adult offender’s age at release is inversely related to the risk of sexual recidivism (e.g., Craig, 2008; Wollert et al., 2010), an association that is routinely considered in constructing risk assessment scales (e.g., Barbaree et al., 2007, 2009). Empirical estimates indicate that the risk of sexual recidivism decreases by 2% to 3% yearly (e.g., Lussier & Healey, 2009). Such observations have had a significant impact on risk assessment practices by indicating the importance of adjusting risk probabilities downward for older adults (e.g., Wollert et al., 2010), even for individuals considered to be at a high risk of sexual recidivism (e.g., Hanson et al., 2014). The well-documented age effect found in recidivism research indicates that, over time and at different paces, all individuals eventually desist from sexual offending. As people age, criminal behavior tends to become less likely for a number of reasons, including maturity, the costs resulting from an arrest/conviction, cognitive transformations, family support, access to new social roles, and therapy (e.g., Lussier, 2016). The resulting desistance narrative emerged from research examining how individuals and their context can change over a life course in such a way as to facilitate the termination of offending (e.g., Farmer et al., 2016; Harris, 2014). Desistance research emphasizes the dynamic aspect of human lives, including the lives of those who have histories of sexual offending, and suggests that the risk of recidivism, risk factors, and responsivity to intervention can change throughout a life course (e.g., Amirault & Lussier, 2011; Mann et al., 2010). These ideas challenge the assumption that individuals with histories of sexual offending remain at risk of sexually reoffending for the remainder of their lives. Recent research has shown that sexual recidivism is unlikely among individuals who have been in the community for 10 to 15 years without a sexual recidivism event (Thornton et al., 2021), at least no more likely to than the general population (Lee et al., 2023), raising significant doubts about the idea that recidivism rates increase linearly as the follow-up period is extended.

These different narratives demonstrate that neither the base rate of sexual recidivism for time-specific periods nor how risk probabilities evolve over time has been determined. Such uncertainty raises important questions for both research and risk assessment. While the criminal justice system pressures professionals to establish long-term risk probabilities that can guide decision-making (e.g., O’Donohue & Bromberg, 2019), sexual recidivism rates for extended follow-up periods are relatively unknown because most studies are based on short-term estimation of risk that covers 5 years or less. It has, however, been suggested that long-term risk probabilities can be predicted using the “constant multiplier assumption,” which proposes that long-term risk probabilities are approximately equivalent to 5-year sexual recidivism risk probabilities multiplied by a factor of about 2 (e.g., Doren, 2010) and can be extrapolated to predict long-term risk estimates.^2^ This approach has been criticized on the grounds that there is too much variability across studies and in actuarial risk assessment methods to allow such simple generalizations. It also fails to consider the age of the person at the time they are released back into the community and the potential that annual probabilities of risk decline over time. As well, recent studies have shown that sexual recidivism rates in Canada and the United States have been declining since the 1970s (Lussier & McCuish, 2024; Lussier et al., 2023). Wollert & Cramer (2012) have urged researchers to collect and analyze empirical data about long-term risk of sexual recidivism rather than relying on invalid assumptions and projections. However, others have suggested that long-term risk probabilities can, at least to some extent, be predicted. Thornton et al. (2021) provide some empirical support for the constant multiplier assumption, showing that individuals who demonstrate a moderate risk of sexual recidivism according to the Static-99R have 10- and 20-year recidivism rates of about 1.5 and 2 times the 5-year rate, respectively. Our current study uses a meta-analytical framework to look at whether these findings can be generalized across time and place.

### Aim of Study

Most risk assessors understand the importance of using the base rate of sexual recidivism in real-world risk assessments (e.g., Bar-Hillel, 1980) that provide risk probabilities for justice-involved individuals. These base rates are also an integral part of actuarial risk assessment instruments (e.g., Craig & Beech, 2010). However, the literature on base rates is not only dense but contains seemingly contradictory narratives, which can lead readers to draw very different conclusions. These seemingly contradictory narratives support the idea that base rates can be ambiguous, unreliable, and unstable (Koehler, 1996). One way to introduce some clarity is to statistically adjust recidivism rates by considering the follow-up period during which recidivism events were examined for a particular sample of justice-involved individuals. Research has shown that sexual recidivism is a time-dependent phenomenon but far more needs to be learned about these time-dependent features. It is expected that studies that use a similar follow-up period will produce similar base rates, but to what extent is this correct? It is expected that sexual recidivism rates will rise as the follow-up period is extended, but to what extent?

The first objective of the current study was to examine the base rate of sexual recidivism across short, moderate, and long follow-up periods and determine how homogeneous these rates are. A second objective was to identify the study details associated with different rates of recidivism for similar mean follow-up periods. Variations in study design and their implications for recidivism rates are often overlooked or minimized so sensitivity analyses were performed to examine the stability of estimates of short, moderate, and long-term recidivism rates as related to various study details and criteria. A third objective was to examine the increase in recidivism rates as a function of the length of the follow-up period while adjusting for various study details. A systematic review and meta-analytical approach was used to examine sexual recidivism rates in relation to the length of the follow-up period in findings from 468 Canadian and American studies. This approach makes it possible to consider findings from studies with different methodologies and conducted in different jurisdictions and to statistically control for details that could influence the observed recidivism rates. Risk assessors work on the assumption that the risk probabilities of sexual recidivism for specific periods are consistent. Our study revisits the various narratives of risk probabilities over time, examining weighted pooled recidivism rates for short, moderate, and long-term follow-up periods for justice-involved individuals with sexual offending histories to determine if this assumption of consistency is warranted.

## Methodology

### Systematic Review

A total of 75 search terms were used to capture the term “sex offender” (e.g., sex* offender, sex* aggressor, child molester), and 74 search terms were used to capture the term “recidivism” (e.g., recidivist, rearrest, repeat offender, supervision failure). The selection of terms relied on researchers’ experience and knowledge about the topic; examination of the scientific literature across academic disciplines; consideration of studies from 1940 to 2019; examination of handbooks and encyclopedias on topics such as abnormal psychology, crime, delinquency, law and mental health, and sexual deviance; and examination of existing literature reviews and previously conducted meta-analyses.

The current study does not include studies published after 2019 given that the data extraction started toward the end of 2019. The data coding was conducted during the 2020 to 2023 period, which included pretesting of the coding instruments and all inter-rater agreement analyses. Over 80 databases, from a variety of disciplines, were searched (e.g., Academic Search Premier [EBSCO]; Criminal Justice Abstracts [EBSCO/Proquest]; Embase [Elsevier]; ERIC [EBSCO]; International Bibliography of the Social Sciences [ProQuest]; Law Journal Library [HeinOnline]; Legal Source [EBSCO]. Medline [Ovid]; NCJRS [ProQuest]; PsycInfo [Ovid]; Sociological Abstract [ProQuest]). A library content comparison tool (Gold Rush) was used to determine an optimal list of databases that had minimal overlap. We also searched for unpublished material (i.e., the “gray literature,” i.e., typically not captured by computerized retrieval systems), including government reports, dissertations, conference abstracts and proceedings, and technical or brief reports to funding agencies (e.g., Open gray; ProQuest Dissertations & Theses Global; DART-Europe; WHO MiNDbank; GreyNet International). Additional details about the study methodology have been previously presented (Lussier, Chouinard et al, 2024; Lussier, McCuish et al., 2024; Lussier et al., 2023).

A four-step procedure was used to identify relevant documents. The first step identified all relevant references (*k* = 24,417) in all inspected sources and databases, imported these references into an electronic document to create a list of references, and then removed duplicates. In the second step, research assistants (RAs) examined these documents, focusing on descriptive information (i.e., study title, abstract, executive summary or study highlights, keywords). RAs were instructed to identify documents that potentially included an empirical assessment of recidivism and a training session was conducted in which RAs and the lead author coded the same set of studies until acceptable inter-rater agreement was reached (Kappa coefficient or *k* > .80). Any document that was not a duplicate and was either directly or potentially relevant to the current study was then extracted (*k* = 3,026). The third step involved analyzing these documents to determine their relevance to sexual recidivism. A document considered as relevant had to be available in its entirety. To avoid discarding research that was difficult to obtain, RAs contacted lead authors and co-authors, searched academic research-oriented websites (e.g., Google Scholar, ResearchGate), used a reverse search approach to triangulate study content using other sources, and contacted researchers who might have a copy of the study. RAs then determined whether the study was empirical (e.g., not a narrative review), longitudinal (e.g., sexual recidivism was not based on examination of offenders’ past criminal convictions), and included some measure of recidivism (e.g., number of recidivists, proportion of recidivists, recidivism rate). At the end of this stage, 859 studies were retained. The fourth step involved identifying which of these 859 studies involved samples from individuals located in the United States or Canada. Using information such as the name and location of the institution from which samples of offenders were drawn, it was determined that 468 studies reported empirical information about sexual recidivism and should be included in this meta-analysis.

### Sample

The 468 studies yielded 626 estimations of sexual recidivism rates for 388,994 individuals. As recidivism data were presented in different formats (e.g., number, percentage, rate), it was decided that for consistency the term “recidivism rate” would be used to designate the probability of recidivism within a sample. There are more recidivism rates than studies because studies sometimes disaggregated sexual recidivism rates across different groups (e.g., “child molesters” vs. “rapists”). If a study reported multiple rates using various criteria (e.g., arrest, conviction) or sources of information (e.g., self-report, official data), the highest rate reported was always used in our analysis. Inter-rater agreement on the coding of sexual recidivism rates was done for 141 studies and was relatively good (Intra-class correlation coefficient (ICC) = 0.86; 95% CI =[0.81, 0.90]). The 626 estimations were pooled and weighted based on sample size to avoid potential biases from, for example, studies that focused on small samples of extremely high-risk perpetrators of sex offenses with high recidivism rates (Borenstein et al., 2021). The rates were pooled (see Appendix 1) using the Freeman–Tukey double arcsine transformation^3^ using the *metaprop* and *metapreg* commands (e.g., Nyaga et al., 2014) in Stata (version 17; StataCorp). The Freeman–Tukey double arcsine transformation addresses limitations (e.g., confidence intervals that fall outside the 0–1 range) in other methods (e.g., invariance method, logit transformation) and stabilizes variance in the pooled prevalence estimates (Barendregt et al., 2013) as well as considering the study sample size and the number of sexual recidivists for each study.

### Pooling Studies

One of the goals of this project is to provide a complete overview of the literature on sexual recidivism. Doing this required considering multiple studies that addressed different questions but used the same or a similar dataset. This is problematic for meta-analysis because a key assumption of such analysis is that observations are independent, which may not be the case if estimates of sexual recidivism rely on the same or overlapping samples (e.g., Cheung, 2019). Determining whether samples are independent is difficult because sample participants are anonymous. A registry of study samples was created that included: the country and province/state in which the sample was drawn; the name and location of the institution, program, and setting from which individuals were sampled; the years the sampling took place; and the length of time in the follow-up period. Studies were then regrouped by sample according to this registry. The next step involved identifying the recidivism rate that best represented the sample. To accomplish this, all samples were examined to determine whether, given the information available, they appeared to be based on the same or approximately the same offender sample.^4^ One sexual recidivism rate (or data point) was selected for each of these groups of samples,^5^ based on, in order of importance, (a) larger sample size, (b) longer follow-up period, and (c) greater level of methodological details reported by authors.^6^ At the end of this process, we established that 238 of the 626 data points initially identified were independent data points for sexual recidivism rates based on samples of 196,651 individuals. We compared findings using these independent data points to findings that used the full sample of studies to investigate not only whether the existent literature is biased toward specific conclusions because of an over-reliance on particular data sources but also whether such biases affect study conclusions (e.g., over- or under-estimation of sexual recidivism rates).

### Recidivism

Sexual recidivism details included the jurisdiction in which recidivism was measured (i.e., state/province-wide or countrywide; *k* = .59), as well as the criteria used to measure recidivism (*k* = .81). Over the years, different criteria have been used to determine whether a recidivism event occurred during the follow-up period. The criteria used were (a) new arrest/charge (57.6%); (b) new conviction (23.1%); (c) new incarceration (2.9%); (d) other criteria (e.g., self-reported involvement in a new sexual offense; 7.1%); and (e) unknown criteria (9.2%). If a study ported multiple rates using different criteria (e.g., arrest, conviction) for the same sample, the highest recidivism rate reported was always used.

### Follow-Up Period

All studies included in the meta-analysis were longitudinal, with recidivism defined as the commission of a sexual event after a sexual offense has already occurred. Follow-up, a window period during which recidivism events are measured for research purposes starts when an individual return to the community and ends the day recidivism data are collected (*k* = .71). Not all recidivism studies, however, include such information, which was then coded as missing. The mean follow-up period for all documents retrieved was 76.1 months (*SD* = 50.5; range: 3–336 months) but after removal of duplicates was 66.1 months (*SD* = 50.8) or about 5.5 years.^7^ A total of 29% of all independent studies retrieved did not include the length of the mean follow-up period, a concerning finding given that this information is essential for interpreting a study’s rate of recidivism and placing it in the context of other research. There are no consensus criteria to categorize the length of a follow-up period into categories. Although the length of the follow-up was mainly analyzed as a continuous variable, it was also categorized for descriptive purposes to conduct the sensitivity analyses. Quartiles were used to create time categories while the last quartile (96 months or more) was split at 144 months (or 12 years) to separate studies with a longer follow-up period, recognizing that such studies are not common in the field.

### Methodological Details of Recidivism Studies

Various study details were measured and statistically controlled to examine whether the prevalence of, and trends in, sexual recidivism rates were artifacts of research design. Senior members of the research team created a coding instrument based on their knowledge of the scientific literature and experience in conducting research on recidivism, as well as examination of sex offender recidivism research in various decades, beginning with the 1940s. Three independent coders pre-tested the instrument on 40 randomly selected studies. Adjustments were then made to solve coding issues and the revised coding instrument was tested on 150 randomly selected publications from a larger pool of international studies. Inter-rater agreement was computed for study details, including publication details, sample characteristics, and recidivism details. Publication details included the type of publication (e.g., peer-reviewed journal; *k* = .94), the lead author’s affiliation (*k* = .80), and the year of publication (*k* = .96). Sample characteristics included the country where the study was conducted (*k* = .93), the study period or the year sampling started (*k* = .99), the study setting (*k* = .67), the age group of the study sample (*k* = .65), and study sample size (*k* = .85). Two additional indicators were added to the study based on a preliminary analysis of the data: lack of clarity about the methodology used and the presence of outliers. To our knowledge, a methodological rigor tool to evaluate sexual recidivism studies does not exist. This is a significant gap in this field of research given the wide variability in research design. In the absence of such a tool, two alternative strategies were taken. First, we collected detailed information about each study’s methodology and controlled for this information in subsequent analyses. Second, an indicator representing the clarity of the study methodology was included. Lack of clarity refers to incomplete information about at least one of the following: the study period or the years during which the sampling was conducted, the measure of recidivism used, the study setting. The outliers (extreme scores with respect to sexual recidivism rates observed) were identified using Galbraith plots (Galbraith, 1988).

### Analytical Strategy

Analyses of study details and their impact on recidivism rates for specific follow-up periods were conducted via random effect models using Stata 18.0 (StataCorp LLC). Random effect models allow for the possibility that random differences between studies are not limited to sampling error, such as variation in settings, procedures, or measurements. This assumption is reasonable given the variability in how recidivism is determined in various jurisdictions (e.g., crime reported to the police, police investigation into events, evidence for events available, event results in court appearance). Inconsistency of pooled estimates was examined using *Q*-statistic (Hedges & Olkin, 1985) and *I*^2^ (Higgins & Thompson, 2002). The *Q*-statistic examined the null hypothesis that the studies are observing the same recidivism rate. The *I*^2^ test statistic, which is based on the *Q*-statistic, provides an estimation of the proportion of total variation across studies that is due to heterogeneity rather than chance. The value of *I*^2^ ranges from 0% to 100% with larger values indicating greater heterogeneity (Higgins et al., 2003).

## Results

**Figure 1.**
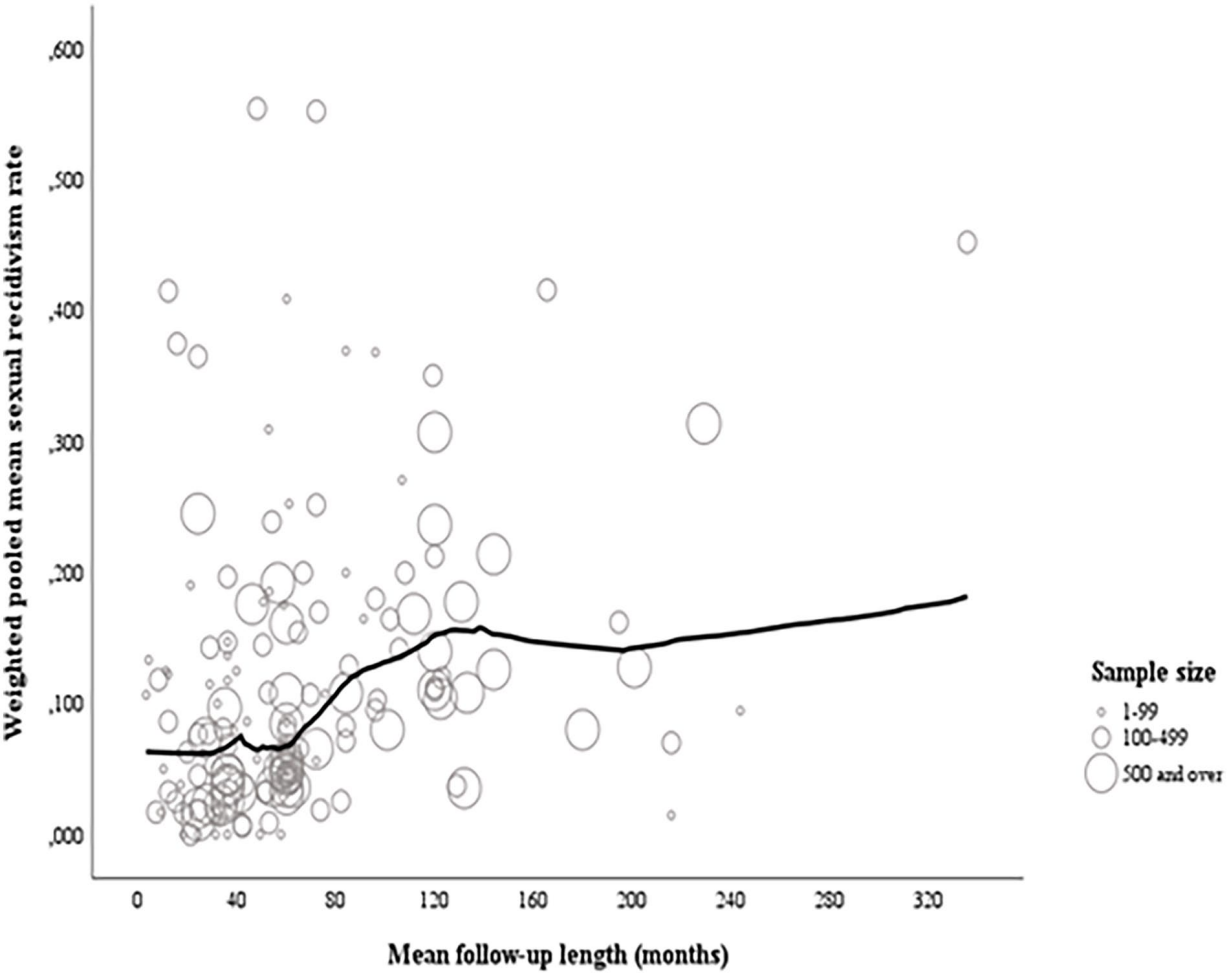
Weighted pooled mean sexual recidivism by the study mean follow-up period. *Note.* Each data point or recidivism rate is represented by a circle. The larger the circle, the larger the sample size and the weight on the trend line computed. Only independent observations are represented (*k* = 238). LOESS trend line was fitted to the data points. LOESS = locally estimated scatterplot smoothing.

Figure 1 shows the weighted pooled sexual recidivism rates by mean length of the follow-up period (months). A locally estimated scatterplot smoothing trend line was fitted to data points for the weighted average rate to highlight its movement as the follow-up period is extended. The trend line can be broken into a series of segments, with the first two segments representing studies with the shortest mean follow-up periods, approximately 60 months. These studies look at recidivism over a very short (less than <36 months) period and have a short follow-up period (at least 36 but less than <60 months). The lowest recidivism rates are found in these two segments and show a very modest increase in the moving average as the follow-up is extended, up to approximately 5 years. That subtle increase starts around a mean follow-up period of 36 months. The next two segments represent sexual recidivism rates over a moderate follow-up period and include studies with a mean follow-up period ranging from 60 months to less than 144 months, or about 12 years. That segment appears critical, given the increase in weighted pooled sexual recidivism rates as the follow-up is extended. The increase can be divided into two segments, with the first from between 60 to about 95 months, and the second between 96 and just shy of 144 months. The final and fifth segment includes studies that provide long-term sexual recidivism rates (144 months and over) and is characterized by a very slow and gradual increase of weighted sexual recidivism. That segment, which starts around the 12th year, has fewer data points as the mean follow-up period is extended. Looking at the association between recidivism rates and the length of the follow-up period highlights two key points. First, recidivism rates do not appear to increase linearly over longer follow-up periods. Second, studies with short, moderate, and long follow-up periods may have unique methodological characteristics that directly impact observed recidivism rates. For exploratory and descriptive purposes, studies were grouped into a continuum of five mean follow-up categories (in months) that were based on the above-mentioned observed segments: (a) less than 36; (b) at least 36 but less than 60; (c) at least 60 but less than 96; (d) at least 96 but less than 144; and (e) at least 144 or more.

Table 1 presents weighted pooled sexual recidivism rates for different mean follow-up periods. Results are given for both non-independent (*K* = 626) and independent samples (*K* = 238) to highlight possible biases due to the over-representation of certain samples in the literature. Not surprisingly, sexual recidivism rates for both non-independent and dependent samples increase as the follow-up period is extended. For non-independent samples, the weighted pooled sexual recidivism rates increased from 0.07 (less than 36 months; 95% CI = [0.05, 0.08]) to 0.21 (144 months or more; [0.18, 0.24]). For independent samples, the weighted pooled sexual recidivism rates increased from 0.06 (less than 36 months; [0.05, 0.09]) to 0.17 (144 months or more; [0.12, 0.23]). When looking at independent samples (*K* = 238), the weighted pooled sexual recidivism rate for the 36 to less than 60 months period (weighted mean = 0.08; [0.06, 0.10]) is roughly half the rate observed for those with a mean follow-up of at least 144 months (weighted mean = 0.17; [0.12, 0.23]).

**Table 1.** Pooled Weighted Mean Rate of Sexual Recidivism According to the Mean Follow-Up Period.

Mean Follow-Up Period (Months)	Non-Independent Samples (*K* = 626)					Independent Samples (*K* = 238)				
	*K*	*n*	Mean	95% CI	*I* ^2^	*K*	*n*	Mean	*SE*	*I* ^2^
Less than 36	78	28,795	0.07	0.05–0.08	95.6	42	26,619	0.06	0.05–0.09	96.5
36 to less than 60	101	48,919	0.07	0.06–0.09	93.1	40	19,394	0.08	0.06–0.10	95.9
60 to less than 96	146	99,351	0.10	0.09–0.11	95.1	46	57,038	0.09	0.07–0.11	96.7
96 to less than 144	82	51,855	0.14	0.13–0.16	96.6	24	17,300	0.15	0.12–0.18	96.5
144 or more	40	12,252	0.21	0.18–0.24	94.7	11	6,560	0.17	0.12–0.23	97.2
Unknown	179	147,821	0.10	0.09–0.11	97.7	75	69,740	0.08	0.06–-0.10	97.8
Test of heterogeneity across follow-up periods	χ^2^(5) = 108.73, *p* < .001					χ^2^(5) = 30.7, *p* < .001				

*Note.* *K* refers to the total number of studies. *N* refers to the total number of individuals included in the studies combined. SE = standard error.

Heterogeneity across follow-up periods was statistically significant for both non-independent and independent samples (*p* < .001). The rates observed per period suggest that in the first 3 years following community re-entry, the annual rate of new sexual recidivism events is about 2%, which then drops to about 1%. However, caution is needed when examining weighted pooled mean sexual recidivism across periods given that the risk probabilities across different time points are based on different studies and samples. There was also significant heterogeneity in each follow-up period as shown by the *I*^2^ coefficients, which are all above 90%. Comparing non-independent and independent samples suggests that over-representation of certain samples in the literature may have created potential biases for long-term estimations of risk. While 40 long-term estimates (at least 12 years) of sexual recidivism were retrieved, only 11 of those were based on independent samples. After duplicates are removed, the mean sexual recidivism rate for 12 years or more drops from 0.21 (95% CI [0.18, 0.24]) to 0.17 [0.12, 0.23]. This means that (a) researchers are likely to reuse longer-term studies for multiple research purposes and (b) the longer-term studies most likely to be reused are those with a higher sexual recidivism rate. This is not surprising given that the longer term makes it possible for researchers to examine multiple long-term issues, such as treatment effect, risk factors, risk assessment tools, etc. It is also possible that longer-term studies tend to be reused not so much because of their higher base rates, but because those are more methodological sound and researchers will invest more time and resources following up such a sample in that context. Although researchers may be reusing these studies simply because they prefer to analyze data with higher base rates of the phenomenon of interest, a collateral consequence may be to suggest that sexual recidivism base rates are higher than what is typical. Such a bias was not observed for studies with a shorter mean follow-up period. In fact, for all estimates, the 95% confidence intervals between independent and non-independent observations overlapped.

A series of sensitivity analyses were performed on independent samples to examine the stability of sexual recidivism rates for specific follow-up periods. Sensitivity analyses to determine whether methodological details affected the weighted pooled recidivism rate observed (Borenstein et al., 2021) were conducted by investigating the stability of recidivism rates for a subset of studies based on specific criterion. Table 2 the lists 26 criteria examined and the recidivism rates observed for each of the follow-up periods considered. Recidivism rates were relatively stable irrespective of the criterion considered, although there were some notable exceptions. For instance, the weighted pooled recidivism rates in documents that were not peer-reviewed (e.g., government reports, PhD dissertations, book chapters) did not increase as the follow-up period was extended. This raises concerns about the methodological rigor and/or the methodological choices of these studies. Studies conducted in a mental health setting (e.g., state hospitals) showed somewhat higher recidivism rates than correctional-based studies, especially for follow-up periods of less than 5 years. After the 3-year mark, countrywide measures of recidivism tend to produce higher recidivism rates than state/province-wide measures, but 95% confidence intervals overlapped across follow-up periods. Removing outlier studies did not significantly change the mean recidivism rates observed. Of importance, recidivism rates were not stable across study periods (i.e., the period when sampling was conducted), even when comparing similar mean follow-up periods. For example, the weighted pooled recidivism rate for older studies (1940–1979) is 0.12, which drops to 0.05 and 0.03 for studies in the 1980 to 1999 and 2000 to 2019 periods, respectively. That drop was not found in studies with a follow-up period of at least 12 years, but this could be due to the small number of such studies. Another way of examining the findings is to examine the time gap to reach similar cumulative sexual recidivism rate across study periods. For example, for the 1940 to 1979 studies, a weighted pooled recidivism rate of 0.15 was reached between 36 and 60 months. For the 1980 to 1999 studies, that weighted pooled rates was reached only for those with a mean follow-up ranging between 96 and 144 months.

**Table 2.** Sensitivity Analysis of the Base Rate of Sexual Recidivism According to the Mean Follow-Up Period.

Number	Criteria	Study Mean Follow-Up Period (in Months)									
		Less Than 36 Months		36 to Less Than 60 Months		60 to Less Than 96 Months		96 to Less Than 144 Months		144 Months or More	
		*K*	Mean [95% CI]	*K*	Mean [95% CI]	*K*	Mean [95% CI]	*K*	Mean [95% CI]	*K*	Mean [95% CI]
1.	U.S. data	37	0.06 [0.04, 0.09]	25	0.07 [0.04, 0.09]	38	0.09 [0.07, 0.10]	14	0.14 [0.10, 0.19]	5	0.12 [0.06, 0.19]
2.	Canada data	5	0.06 [0.04, 0.09]	15	0.10 [0.06, 0.15]	8	0.11 [0.08, 0.15]	10	0.16 [0.12, 0.20]	5	0.24 [0.15, 0.35]
3.	Peer-reviewed article	28	0.06 [0.04, 0.10]	30	0.09 [0.06, 0.13]	31	0.09 [0.07, 0.12]	23	0.15 [0.12, 0.19]	10	0.18 [0.12, 0.25]
4.	Other documents (e.g., government report)	14	0.06 [0.04, 0.10]	10	0.04 [0.03, 0.06]	15	0.08 [0.06, 0.11]	1	0.11 [0.09, 0.13]	1	0.08 [0.07, 0.10]
5.	Lead author’s affiliation (university)	23	0.07 [0.03, 0.12]	19	0.09 [0.04, 0.14]	30	0.09 [0.07, 0.12]	14	0.15 [0.11, 0.20]	9	0.18 [0.12, 0.26]
6.	Sample size ≥ 200	13	0.04 [0.03, 0.06]	17	0.06 [0.04, 0.09]	27	0.08 [0.06, 0.10]	18	0.14 [0.11, 0.18]	6	0.15 [0.09, 0.21]
**7.**	Sample size ≥ 500	7	0.05 [0.03, 0.09]	11	0.06 [0.04, 0.08]	13	0.06 [0.05, 0.08]	10	0.14 [0.10, 0.19]	5	0.16 [0.10, 0.24]
8.	Publication year (≥2000)	25	0.05 [0.03, 0.07]	35	0.08 [0.06, 0.10]	37	0.09 [0.07, 0.11]	21	0.14 [0.11, 0.17]	10	0.15 [0.10, 0.21]
9.	Publication year (≥2010)	17	0.04 [0.02, 0.08]	24	0.08 [0.05, 0.12]	24	0.08 [0.06, 0.10]	12	0.14 [0.09, 0.19]	6	0.14 [0.08, 0.20]
10.	Sampling period (1940–1979)	8	0.12 [0.04, 0.23]	8	0.15 [0.09, 0.21]	4	0.10 [0.04, 0.18]	5	0.21 [0.12, 0.32]	3	0.15 [0.09, 0.17]
11.	Sampling period (1980–1999)	9	0.05 [0.03, 0.09]	15	0.07 [0.04, 0.11]	26	0.09 [0.07, 0.11]	16	0.14 [0.10, 0.17]	7	0.18 [0.11, 0.26]
12.	Sampling period (2000–2019)	17	0.03 [0.02, 0.06]	18	0.08 [0.05, 0.11]	14	0.09 [0.05, 0.14]	2	0.11 [0.09, 0.13]	—	—
13.	Clarity about the methodology used	32	0.06 [0.04, 0.08]	33	0.08 [0.05, 0.10]	41	0.09 [0.07, 0.11]	21	0.15 [0.12, 0.19]	10	0.17 [0.11, 0.25]
14.	Lack of clarity regarding the methodology	10	0.10 [0.04, 0.19]	7	0.10 [0.04, 0.18]	5	0.07 [0.04, 0.11]	3	0.13 [0.10, 0.17]	1	0.13 [0.11, 0.15]
15.	Correctional-based setting	17	0.05 [0.03, 0.08]	23	0.06 [0.04, 0.08]	28	0.08 [0.06, 0.10]	7	0.12 [0.09, 0.16]	6	0.20 [0.12, 0.29]
16.	Prison-based study	7	0.03 [0.01, 0.07]	13	0.04 [0.03, 0.06]	17	0.08 [0.06, 0.10]	7	0.14 [0.11, 0.18]	5	0.26 [0.14, 0.39]
17.	Mental health setting	10	0.08 [0.03, 0.14]	7	0.11 [0.05, 0.19]	5	0.14 [0.05, 0.27]	10	0.15 [0.11, 0.20]	2	0.21 [0.19, 0.23]
18.	Juvenile justice setting	7	0.06 [0.03, 0.10]	6	0.06 [0.01, 0.13]	10	0.07 [0.05, 0.09]	6	0.15 [0.08, 0.24]	1	0.16 [0.11, 0.23]
19.	Adult sample	26	0.06 [0.04, 0.09]	28	0.08 [0.06, 0.11]	30	0.10 [0.07, 0.12]	13	0.14 [0.11, 0.18]	7	0.18 [0.11, 0.25]
20.	Males only sample	27	0.07 [0.04, 0.10]	28	0.08 [0.05, 0.12]	33	0.09 [0.07, 0.11]	20	0.15 [0.12, 0.19]	5	0.20 [0.11, 0.32]
21.	General sample of offenders	30	0.06 [0.04, 0.09]	29	0.07 [0.05, 0.09]	30	0.08 [0.06, 0.19]	14	0.17 [0.13, 0.22]	8	0.18 [0.12, 0.25]
22.	Countrywide measure of recidivism	7	0.04 [0.02, 0.08]	14	0.09 [0.06, 0.14]	12	0.11 [0.07, 0.16]	13	0.18 [0.14, 0.23]	5	0.24 [0.15, 0.35]
23.	Province/statewide measure of recidivism	35	0.07 [0.05, 0.10]	26	0.07 [0.05, 0.10]	34	0.08 [0.07, 0.10]	11	0.11 [0.08, 0.15]	6	0.12 [0.06, 0.19]
24.	Recidivism as a new arrest/charge	27	0.06 [0.03, 0.09]	22	0.07 [0.05, 0.09]	28	0.07 [0.06, 0.09]	17	0.14 [0.10, 0.18]	7	0.14 [0.08, 0.22]
25.	Recidivism as a new conviction	7	0.04 [0.02, 0.06]	11	0.06 [0.03, 0.10]	12	0.09 [0.07, 0.13]	5	0.19 [0.11, 0.30]	4	0.22 [0.11, 0.36]
26.	Removing outliers	38	0.04 [0.03, 0.05]	38	0.07 [0.05, 0.08]	43	0.08 [0.06, 0.09]	23	0.14 [0.11, 0.17]	8	0.17 [0.11, 0.23]

*Note*. Analyses were conducted using independent samples only. Outliers were identified using Galbraith plot by examining data by follow-up periods. Data points falling outside the 95% confidence intervals were removed from the analyses and the weighted pooled recidivism was re-examined. The number of outlier studies (*K* = 12; 5%) was as follows: (a) less than 3 years (*K* = 4); (b) between 3 and less than 5 years (*K* = 2); (c) between 5 and less than 8 years (*K* = 3); (d) between 8 and less than 12 years (*K* = 1); (e) at least 12 years (*K* = 2). There were not enough female-only independent samples to run the analyses by length of follow-up periods.

Next, a series of meta-regressions were conducted to examine the association between mean follow-up period and sexual recidivism rates while statistically adjusting for certain methodological details (Table 3). Only studies that provided information on the follow-up were included (*K* = 163). Linear, quadratic, and cubic functions for follow-up period (in months) were included in all analyses. Given the results reported earlier, the study period was considered in both meta-regression models tested, with the study period 1940 to 1979 used as the reference category. All covariates were statistically significant. Model 1 highlights a significant drop for the 1980 to 1999 period and a more pronounced drop for studies whose sampling took place during the 2000 to 2019 period. The quadratic and cubic functions improved the fit between sexual recidivism rates and the mean length of the follow-up period. To challenge these findings and verify that under-fitting had been avoided, Model 2 includes the country in which the study was conducted, study setting, sample size, clarity about methodology, whether recidivism was measured based on arrest data (e.g., as opposed to conviction data), and whether recidivism was measured country-wide (e.g., as opposed to state-level). As the mean length of the follow-up period was extended, sexual recidivism rates increased, even after adjusting for multiple covariates and possible confounding factors. The cubic function of the mean follow-up period became marginally significant (*p* = .052) but otherwise, the findings remained relatively stable. Among the new covariates added to Model 2, studies reporting clear information about the methodology used reported higher recidivism rates (*p* = .024). Studies using arrest/new charge as a criterion reported significantly lower rates than those using another measure of recidivism (*p* = .049).

**Table 3.** Meta-Regression Analyses of the Covariates of Weighted Pooled Mean Sexual Recidivism Rate.

Covariates	Model I				Model II			
	Coefficient	*SE*	*Z*	*p* Value	Coefficient	*SE*	*Z*	*p* Value
Study period^a^								
1980–1999	−0.147	0.057	−2.57	.010	−0.158	0.063	−2.51	.012
2000–2019	−0.200	0.060	−3.35	.001	−0.204	0.067	−3.08	.002
Unknown	0.025	0.077	0.32	.748	0.152	0.102	1.48	.139
Mean length of the follow-up period (months)								
Linear	0.006	0.002	3.21	.001	0.006	0.002	3.01	.003
Quadratic	−0.000	0.000	−2.25	.025	−0.000	0.000	−2.17	.030
Cubic	0.000	0.000	2.06	.039	0.000	0.000	1.95	.052
Country								
Canada					0.073	0.052	1.39	.166
Study setting								
Mental health hospital					0.028	0.049	0.58	.559
Prison-based study					−0.046	0.042	−1.11	.268
Sample								
Sample size					−0.000	0.000	−1.71	.087
Study design								
Clarity about the methodology					0.186	0.083	2.25	.024
Measurement of recidivism								
Arrest/charge					−0.076	0.039	−1.97	.049
Countrywide measure					0.031	0.048	0.65	.515
Constant	0.520	0.076	6.87	.000	0.398	0.106	3.77	.000
Model fit	Wald χ^2^(6) = 58.30, *p* < .001				Wald χ^2^(13) = 74.80, *p* < .001			

*Note*. *K* = 163. Analyses conducted using only independent observations. Only studies with a known mean follow-up period were included in these analyses.

a The reference category is 1940 to 1979.

## Discussion

Earlier empirical studies reported that cumulative recidivism probabilities increase over time and the current study findings reinforce the importance of considering this time component (e.g., Hanson et al., 2003; Lussier, Chouinard et al., 2024; Soothill & Gibbens, 1978; Thornton et al., 2021). Therefore, placing recidivism rates in the context of the length of the follow-up period is critical for communicating risk probabilities. While this statement might appear obvious to most researchers, 30% of the studies retrieved in the systematic review did not report information about the length of the follow-up period. As a whole, reported rates of sexual recidivism vary widely across studies. This is concerning because it can lead to vast disparities, for example, in how the risk of recidivism is communicated to courts, parole boards, and the general public. Such information about time is critical because it provides context for sexual recidivism rates. Short-term risk is not necessarily a reflection of long-term risk. For example, if a sample’s probability of recidivism over a 5-year period is .10, it is inaccurate to conclude that this same sample’s probability of recidivism over a 10-year period is .20. The literature often fails to recognize that risk is a dynamic, time-dependent phenomenon and even when the temporal component of recidivism is acknowledged, less attention is given to the functional form of the relationship between probability of recidivism and follow-up length. The current study’s systematic review and meta-analysis of 468 studies fills this gap by identifying weighted pooled sexual recidivism for various mean follow-up periods, including the weighted pooled mean recidivism rate for the very short period of less than 36 months (Mean = 0.06; 95% CI [0.05, 0.09]), roughly about 2% to 3% annually. This is consistent with prior findings showing that yearly recidivism rates are higher in the first few years following community re-entry (Hanson et al., 2014). When the mean follow-up period is extended to at least 60 months (but less than 95 months), mean sexual recidivism rates increased to 0.09 [0.07, 0.11]. This corresponds to a yearly rate of increase of about 1% to 2%. For long-term studies with a mean follow-up period of at least 144 months, the weighted pooled sexual recidivism rate found was 0.17 [0.12, 0.23], which highlights the increase in rate as the follow-up period is extended. In studies with follow-up periods longer than 144 months, the yearly sexual recidivism rate drops to about 1%.

The observed pattern of sexual recidivism rates across different mean follow-up periods has at least three implications for case formulation and risk management. First, while recidivism studies with short-term follow-up periods report the lowest sexual recidivism rates, the yearly hazard rate appears to be the highest. This suggests that the initial period which marks the start of the follow-up period (e.g., community re-entry) may be critical from a risk-management standpoint for justice-involved individuals at a high risk of sexual recidivism (e.g., Lussier & Gress, 2014). Second, weighted pooled sexual recidivism rates do not increase in a linear fashion as the follow-up period is extended, a finding that is consistent with earlier conclusions that the likelihood of sexual recidivism decreases with time and aging (e.g., Barbaree et al., 2009; Lussier & Healey, 2009; Wollert et al., 2010). Our study provides support for Thornton et al. (2021)’s findings that the 10-year sexual recidivism rate is about 1.5 times the 5-year rate. For example, for a mean follow-up period ranging between 96 and 144 months, the weighted pooled sexual recidivism rate was 0.15, which is 1.67 times the rate found for a period ranging between 60 and 96 months. This seems to support the idea that there is a constant multiplier effect at play (e.g., Doren, 2010). Third, the idea that individuals always remain at risk of sexual recidivism is not supported by our findings, which suggest that sexual recidivism rates after a mean follow-up of 120 months (see Figure 1) continue to increase but only very slowly. These findings are in line with the idea that individuals who have been offense-free for a 10-year period are unlikely to sexually reoffend (e.g., Hanson, 2018; Lee et al., 2023).

Some discrepancies between American and Canadian studies highlight the importance of international comparison on recidivism rates, a relatively unaddressed topic in recidivism research (e.g., Lussier et al., 2023; Redondo et al., 1999; Yukhnenko et al., 2023). Recidivism research relies heavily on official sources of information on offending, whether a new arrest or a new conviction, which are indirect measures of the functioning of the criminal justice system. International comparisons, therefore, are bound to historical, political, social, legal, judicial, criminological, and methodological issues (e.g., Aebi, 2010; Van Dijk et al., 2021; Xie & Baumer, 2019) which impact recidivism research and the estimation of recidivism rates (Lussier et al., 2023). For example, our initial findings suggest that, over time as the follow-up is extended beyond 12 years, sexual recidivism rates tend to increase in Canada, whereas in the United States, they tend to stabilize. It is reasonable, from a practical standpoint, to raise the question as to whether it is advisable to combine American and Canadian data in order to avoid creating biases due to international differences.^8^ For several reasons, differences observed in the present study are difficult to interpret based on the information available. Indeed, differences observed in long-term rates might be more apparent than real, and these findings should be interpreted with caution. The limited number of studies with a follow-up period of at least 12 years raises questions as to the reliability of *both* American and Canadian estimates. This point is particularly important given that the 95% confidence intervals of American and Canadian weighted pooled sexual recidivism rates overlap (U.S.: [0.06, 0.19]; CAN: [0.15, 0.35]). Furthermore, the difference in long-term sexual recidivism rates (U.S. = 0.12; CAN = 0.24) should also be considered in terms of the range of the 95% confidence interval for this difference is [0.00, 0.24]. To determine whether a recidivism event occurred, American studies tend to be limited to state-level data whereas Canadian studies tend to rely on centralized police records and national-level data. In the United States, inconsistencies between state-level and national-level data and the complexity of obtaining and using national-level data might explain decisions made in American studies to rely on state-level data, despite the limitations implied (e.g., Durose et al., 2019). Findings of the meta-regression show that while accounting for such methodological discrepancies between state-level and national-level data, differences between Canada and the United States were not statistically significant (see Table 3). In all, these findings emphasize the need for more studies including a long-term follow-up period across different jurisdictions including both state/province-level as well as national-level data.

Before concluding that a 10-year period free of sexual reoffending is used as a consistent benchmark to determine that a person’s risk of sexual recidivism is low, such a finding needs to be observed consistently across a wide range of risk-assessment contexts.^9^ In other words, among the small number of long-term follow-up studies, is there variability in reported sexual recidivism rates, and is this variability tied to the methodological details of such studies? While our study findings provide risk probabilities for different time points, they also reflect the differing nature, context, and methodological characteristics of studies. Sensitivity analyses provided some clues about the source of variations in methodological choices and details, with two sources raising concerns about aggregate sexual recidivism rates for specific time periods. First, when sexual recidivism rates for studies with a comparable mean follow-up period were pooled, sexual recidivism rates were significantly heterogeneous. For example, weighted pooled sexual recidivism rates across studies with a mean follow-up period of less than 36 months varied between 0.03 (prison-based studies) and 0.12 (studies whose sampling was conducted between 1940 and 1979). Variability was even more pronounced for studies with a long follow-up period: the weighted pooled sexual recidivism rate for studies with a mean follow-up period of at least 144 months ranged from 0.08 (documents that were not peer-reviewed scientific articles) to 0.26 (prison-based studies). Second, we found that the recidivism rate function diverged across sensitivity analyses. For example, the overall mean sexual recidivism rate for a mean follow-up period of at least 144 months was 1.89 times that of the sexual recidivism rate for a period ranging between 60 and 96 months. When rates for these two periods were compared across our sensitivity analyses, the constant multiplier varied between 1.00 (documents other than peer-reviewed articles) and 3.25 (prison-based studies). Such significant variation suggests that the evolution of sexual recidivism rates over time depends on the context and methodological rigor of studies. Our findings show that (a) recidivism rates can vary significantly in different contexts, even for a similar follow-up period and (b) the function of the sexual recidivism rate over time can vary significantly depending on context and methodological details, demonstrating that there is no single rate or formula that characterizes sexual recidivism rates over time because such rates vary in relation to context, person, place, period, and methodology.

### Study Limitations

The examination of sexual recidivism rates for specific follow-up periods is hampered by several conceptual and methodological issues. For example, there is no consensus as to what is a short, moderate, or long-term recidivism rate. The rate of recidivism for a given mean follow-up period is not exactly the same as the rate for a specific period in which all members of a cohort are followed for the same period. Furthermore, while a significant number of studies did not report the length of the follow-up period, even most studies that reported it made no mention of the “time at-risk.” In contrast to “length of the follow-up period,” the time at-risk considers all periods during which individuals were not in the community (e.g., incarcerated for a nonsexual offense) and/or could not perpetrate a sexual offense for some reason (e.g., deported, deceased). Furthermore, a sample was used only once in calculating recidivism rates based on the mean follow-up period for that particular sample. In other words, if a study had a mean 10-year follow-up period, that sample was not considered for the computation of recidivism rates for shorter periods (e.g., less than 3 years). It is difficult to determine the effect of researchers’ failure to report sexual recidivism rates for various follow-up periods on the weighted pooled sexual recidivism rates for different mean follow-up periods. The current meta-analysis is also limited by the information provided by researchers—there are factors not measured in these studies that might have impacted the recidivism rates observed. Finally, the limited number of studies reporting sexual recidivism rates for a very long follow-up period (20 years or more) highlight the issues and difficulties involved in conducting such research (e.g., tracking a cohort of individuals over two decades). More research is needed to clarify the long-term risk of recidivism.

## Conclusion

The criminal justice system regularly pressures professionals to forecast the risk of sexual recidivism by justice-involved individuals over a 5-, 10-, or 20-year period. Such forecasting has become routine in criminal justice practices in response to the laws, policies, and programs aimed at preventing individuals from sexually reoffending (e.g., Sreenivasan et al., 2010). This forecasting is often informed by longitudinal research that examines sexual recidivism rates for specific follow-up periods. It is unclear whether this field of research has produced, in spite of 468 publications on this issue, detailed information that can be used in highly specific contexts. In the absence of clear guidelines as to how to conduct such research and report such findings, we are left with more questions than answers. For example, not only a considerable number of studies did not include the length of the follow-up period, the vast majority of researchers did not report the time at-risk (or exposure time) which considers periods during which individuals were hospitalized, incarcerated (for a nonsexual offense), emigrated to a different country, had passed away or other reasons that could impact their ability to sexually reoffend in a specific time and place where recidivism data were collected (e.g., Berg, & Huebner, 2011; Skardhamar & Telle, 2012). For the field to move forward, guidelines should be set with respect to the operationalization of the time component of recidivism, which should include: the study sampling start date and end date, the study start and end date of the follow-up period, the length of the follow-up period and how it was determined, whether exposure time was examined and statistically controlled for, whether authors determined if justice-involved individuals were alive throughout the follow-up period, etc. Such guidelines are pivotal and important because: (a) more detailed information will allow readers/users of the research to accurately contextualize the findings and better communicate risk-relevant issues; (b) this will allow researchers to make more accurate comparisons between their findings and those of prior studies; and (c) this will also help to increase standardization in a field of research that desperately needs it (see also, Singh et al., 2015).

Research publications and risk assessment tools provide benchmarks to assist in such risk assessment, but these benchmarks tend to be based on a single study conducted in a particular context, in a given time and place, under specific methodological conditions and choices. The current study used a systematic review and meta-analysis framework to compare and contrast sexual recidivism rates across 238 independent samples to provide a more comprehensive overview of sexual recidivism for specific follow-up periods. We did not find evidence to support the view that justice-involved individuals as a group, are at high risk of sexual recidivism (see also, Soothill, 2010), even when the length of the follow-up period is extended beyond 12 years on average. However, there is no consensus as to what “high-risk” means and various operational definitions have been used in research and in legal procedures (Hanson et al., 2024). We found a some heterogeneity in reports of sexual recidivism rates for a similar follow-up period across contexts, settings, and study periods. We also found evidence that the sexual recidivism function increases differently over time across contexts. Such variations highlight the importance of validation and (re-)calibration of risk assessment tools, recognizing that there is no single rate that captures the risk of sexual recidivism for all justice-involved individuals. From a research standpoint, it is important to consider the information provided in more than 80 years of recidivism research in order to improve research practices and standards. Our study provides evidence that variations in research design and methodological rigor are associated with different sexual recidivism rates, which is concerning given the absence of standards and guidelines to how recidivism research should be conducted, how recidivism research findings should be communicated, and how such findings should be used in the criminal justice system.

Helping the field move forward requires increased interest in creating standards and guidelines for recidivism research as well as examining the question of risk, risk assessment, and risk management practices across different contexts and places, for different groups, and under different conditions. The scientific literature on risk, risk assessment, and risk management of justice-involved individuals is over-represented by American research (see Lussier, Chouinard et al., 2024) and given the particularities of the American criminal justice system and its response to perpetrators of sexual offenses, study findings from American studies might not generalize elsewhere. The inclusion of Canadian studies in the current meta-analysis stresses the importance of recognizing that, behind statistical risk probabilities, are embedded a series of under-addressed contextual factors (e.g., political, legal, judicial, correctional; see Maltz, 2001). It is important to keep in mind that sexual recidivism rates are mainly based on information stemming from official sources (e.g., police/court data) and, as a result, partly reflect the functioning of the criminal justice system where the study is conducted. Research examining risk-related issues across a diversity of contexts but also across ethnic, gender, and age groups remains an ongoing issue for this field of research (e.g., Shepherd & Lewis-Fernandez, 2016). While there has been growing research interest for the issue of diversity, it has been limited to issues related risk prediction variation and potential test biases. Researchers have shown that age, sex, and race are statistically associated with recidivism rates, but the factors responsible for such differences remain somewhat elusive (e.g., Piquero et al., 2015). More work is needed to understand the diversity of experiences among justice-involved individuals as they come into the contact of the police, the courts, prison and probation services, treatment programs, and how such experiences relate to recidivism (e.g., Grattet et al., 2011). Research has shown that recidivism rates are influenced by social and neighborhood-level factors (e.g., access to community resources, racial segregation) that might differentially impact certain groups (e.g., Chamberlain & Wallace, 2016; Mears et al., 2008). In the current meta-analysis, there was an insufficient number of studies available to examine time-adjusted sexual recidivism rates across sex, race, and ethnic groups and the current study findings should be interpreted accordingly. For example, only 2.5% of the studies included in the current meta-analysis that reported information about perpetrators’ sex were based on a sample of female perpetrators of sexual offenses. Yet, there is much reason to believe, for example, that the current study findings might not apply to women as prior research as shown their risk of sexual recidivism to be significantly lower than men (e.g., Cortoni et al., 2010). Hopefully, the current study will encourage the research community to pay greater attention to diversity-related issues to help inform professionals making decisions that are better informed.

### Critical Findings

- The current study findings highlight the importance of time in the measurement of recidivism rates.
- Close to 30% of recidivism studies do not report information about the length of the follow-up period, which is a critical information to interpret recidivism rates.
- The current study provides critical information about the base rate of sexual recidivism for short, moderate, and long-term follow-up periods.
- The cumulative sexual recidivism rates vary between 0.06 (or 6%) and 0.17 (or 17%) when statistically controlling for the length of the follow-up period.
- Much heterogeneity in risk probabilities was found even for studies with a similar follow-up period.
- The observed heterogeneity in sexual recidivism rates is partly explained by the methodological features of the study.
- The current study demonstrates the relevance of a quantitative meta-analytical framework to provide some clarity about the cumulative risk of sexual recidivism for short, moderate, and long-term periods.

### Implications of the Review for Practice, Policy, and Research

- The current study findings challenge the common perception that all perpetrators of sexual offenses eventually sexually recidivate and reiterate the importance of risk assessment.
- Risk communication is a critical component of risk assessment practices, which can involve establishing the long-term risk of sexually reoffending.
- Recidivism studies are important for risk assessment purposes to provide potential benchmarks to establish risk probabilities over time.
- Having said this, several issues and challenges were noticed with respect to the measurement of the follow-up period in recidivism studies.
- There are few studies examining with a follow-up period of 20 years or more, which limit drawing firm conclusion about long-term risk.
- Extrapolating long-term risk probabilities from short-term risk probabilities using a constant multiplier is not supported by the study findings.
- The study highlights significant problems and issues with the study of sexual recidivism, but also the reporting of study findings.
- The current study calls for clear guidelines and better standards for recidivism-based research.

## Appendix 1

### Pooling and Weighing the Prevalence of Sexual Recidivists Across Studies

(A1)
$$t=\sin^{-1}\sqrt{\frac{n}{N+1}}+\sin^{-1}\sqrt{\frac{n+1}{N+1}}$$

where *n* refers to the number of cases in the category (recidivist), *N* refers to the total sample size. The variance of *t* can be obtained with the following:

(A2)
$$\mathrm{Var}\left(t\right)=\frac{1}{N+0.5}$$

The transformed values are then transformed back to the original unit of proportions using the following formula:

$$p=\;\frac{1}{2}\;\left[1-\mathrm{sgn}(\cos\;t)\sqrt{\left[1-{\left(\sin t+\frac{\sin t-\frac{1}{\sin t}}{N}\right)}^{2}\right]}\right]$$

## Notes

1. Previous research supporting this first narrative was based on the idea that additional sexual offenses may have occurred in the time between release and a repeat offense being detected and sanctioned, although more recent research suggests that the detection rate might actually increase following an initial sanction (Kelley et al., 2023).

2. For example, if an actuarial risk assessment tool finds that a person has a .35 risk probability of sexual recidivism over a 5-year period, that person would have a .70 risk probability over a 20-year period, making sexual recidivism very likely. If a person has a .05 probability of sexual recidivism over the 5-year period, that future probability of risk would be around .10, suggesting that sexual recidivism is, comparatively speaking, less unlikely.

3. In recent years, the Freeman–Tukey transformation has been somewhat criticized (Lin & Xu, 2020), although more recent studies suggest that such criticism might have been overstated (Doi & Xu, 2021; Lin et al., 2022). It remains one of the most widely used methods for the meta-analysis of proportions (e.g., Monk et al., 2024; Sulaiman et al., 2024).

4. A computerized database was created to organize and classify study samples by country, state/province, and setting (e.g., institution/treatment program). Each sample was given an identification number, and all data points stemming from the same institution/program (i.e., treatment program, penitentiary, etc.) were regrouped to account for sample dependence.

5. It made little sense to statistically pool all findings from the same location/institution (e.g., the same 500 cases analyzed after 1, 3, 10 years in different publications). We considered using multilevel analyses by nesting rates per study location but there was too much variation in methodological features across publications to make this feasible.

6. Due to incomplete information, reading each study’s methodology was not always sufficient to allow us to establish whether different publications were based on overlapping samples. Additional information was therefore collected from other sources (e.g., by examining related publications and contacting authors), which sometimes included making inferences about an author’s affiliation or comparing descriptive sample statistics (e.g., sample size, mean age).

7. The mean length of the follow-up period was associated with various study details: the country where the study was conducted (χ^2^(5) = 20.7, *p* < .001), the study period (χ^2^(15) = 37.3, *p* < .001), whether a countrywide measure of recidivism was used (χ^2^(5) = 13.7, *p* = .012), and the absence of information about the measure of recidivism itself (χ^2^(5) = 12.3, *p* = .031). These show the importance of statistically adjusting rates for study details. American studies were more likely to fail to include information about the follow-up period (35.1% vs. 18.9%) and were less likely to report recidivism rates for short periods (less than 3 years: 20.0% vs. 9.4%) than for longer periods (at least 8 years: 28.3% vs. 10.8%).

8. This is important, for example, when criminal justice professionals rely on an actuarial risk assessment instrument developed using data from a particular jurisdiction and the instrument is then used for risk assessment in another jurisdiction.

9. For example, studies that report that a person did not sexually recidivate over a 10-year period rarely also report whether such persons recidivated with non-sexual offenses. It is possible, for example, that a person who did not sexually recidivate over a 10-year period was involved in a violent offense 2 years after release into the community that subsequently resulted in an 8-year prison sentence. In other words, studies need to be able to answer whether the absence of sexual recidivism was influenced by a person’s involvement in other offenses that resulted in incarceration and a reduced opportunity to sexually recidivate.
